# Supplementary material for: An investigation of the diachronic trend of dependency distance minimization in magazines and news
Source: PLoS One. 2023 Jan 5;18(1):e0279836. doi: 10.1371/journal.pone.0279836 (PMC9815589; doi:10.1371/journal.pone.0279836)
Supplement: Attachment — Submitted filename: Review on the diachronic trend of dependency distance in news and magazines.docx [file pone.0279836.s002.docx]

Review on

**An investigation of the diachronic trend of dependency distance in news and magazines**

This study quantitatively investigates the principle of dependency distance minimization (DDM) based on COCA. With special attention paid to the genres of news and magazines, it was found that both of these two text types witnessed some fluctuations diachronically in terms of the index of MDD and NDD, especially the text type of news. Moreover, the present study suggests that news texts achieve a balance between language complexity and communication efficiency.

Generally, the current work makes some exploration into the diachronic changes of the memory burden of these two text types. I appreciate that the authors make a good explanation of the theoretical background of the research and demonstrate their results clearly and concisely. However, one weak point of the work is the insufficient explanations of the reasons for the fluctuation of MDD and NDD with more references and citations to reach the conclusion that the text type of news cherishes a balance between complexity and efficiency.

Anyway, the article is a typical quantitative linguistic study that brings new findings to the diachronic changes of dependency distance and new insights into how MDD/NDD comes into force on different text types.

**I recommend accepting this study but the comments, which I itemize below, might be taken into consideration.**

**Comments on some specific sections:**

**Title**

- It might be better to change the title from “An investigation of the diachronic trend of dependency distance in **news and magazines**” to “An investigation of the diachronic trend of dependency distance in **magazines and news**”, since in the **Results and Discussion** part, the analysis of magazines are presented first, and then magazines. Also, please check through the whole text the order of “magazines” and “news”, including Tables.

**Abstract**

- Line 10: “inspection indicators of MDD and NDD” > please give the full name of MDD and NDD in the abstract since they are not so familiar to the general readers of PLOSone.

**Section 1 Introduction**

- Line 102: “language, compared with the dependency distance of languages randomly generated” > two spaces between “languages” and “randomly” should be deleted.
- Line 101-159: The author grouped quantitative dependency studies into 7 different types. However, it is not so clear in terms of the differences among so many groups. I recommended dividing this part into different themes. For example, it can be divided into the factors that may affect dependency distance (e.g., Jiang and Liu 2015; Wang and Liu 2017; Yan and Liu 2021), the mechanism of dependency distance minimization and its impact (e.g., Liu 2007; Lu, Xu and Liu 2016), and the application of dependency measures and quantitative features of dependency structures (e.g., Liu, Zhao & Li 2009; Liu and Xu 2012; Jing and Liu 2015). Then, it comes to the lead-in part of the topic of the current research.

**Section 2 Research Methods**

- Line 182-183: change the font of Table 1 to be consistent with the whole text or the format requirement of PLOSone.
- Line 213: add a citation to “Standard CoreNLP 4.4.0”, e.g., Manning et al. 2014 (maybe).
- Line 221-223: “Furthermore, considering the huge volume of the COHA corpus, the author randomly selected 30% of the original corpus for analysis and generated an annotated corpus for data analysis based on the randomly sampled text.” > Add information on the final raw data adopted after the data pre-processing and random selection. A table similar to Table 1 might be favorable.
- Line 224-225: “the average dependency distance MDD and the normalized average dependency distance NDD.” > (1) The expression should be reformulated. e.g., “the average dependency distance (MDD) and the normalized average dependency distance (NDD)”; (2) the citation should be added after MDD (Liu 2008) and NDD (Lei and Jockers 2020).
- Line 228: punctuation mistake (please check through the whole text): *the lazy* *dog,” > dog.”,*
- Line 232: In Table 2, row 1, column 2, “Parent node word(governor)” > “Parent node word (governor)”
- Line 242: “normalized NDD” > the “N” means normalized.
- Line 247: “MDD values can still be relied upon with large data sets”> two spaces between “values” and “can” need to be deleted.
- Line 251: “The corresponding numerical values calculated are significantly” > two spaces between “values” and “calculated” need to be deleted.
- Line 252: “Pearson correlation coefficient” > please check the assumptions of Pearson correlation. If the assumptions are not met, please use Spearman correlation.
- Line 252: 0.922 > please explicit the range of high, medium and low coefficients, respectively.

**Section 3 Results and Discussion**

- Line 267: the present author > the author or the present study
- Line 274: R² > italicize statistics (*R²*)
- Line 280-281: “The goodness of fit of MDD and NDD for magazine texts is high” > what is “high”? Give the value ranges for good, medium and low coefficients of R square.
- Line 320-335: The discussion is interesting and provoke-thinking. However, one very important shortcoming of this part is that more information on the features of news and magazines, especially more explanations of the characteristics of the news text type to reach a balance between syntactic complexity and communication efficiency, is highly needed.

**Section 4 Conclusion**

- Line 341-342: “Not all text types can reflect the diachronic dependency distance minimization trend” > this sentence is a little bit strange. You may delete it or add more explanations to this sentence.
- Line 347: “ the upper limit of the dependence distance as 3” > should it be 4, rather than 3? Please check it and add a citation.
- Line 359: “could be regarded as mass media. [27]” > wrong placement of the punctuation mark, it should be “could be regarded as mass media [27] .”
- Line 359-361: “The research results have revealed that the language complexity of magazines, elite media, has now approached that of mass media.” > add more explanation of this sentence in **Results and Discussion** so as to reach this conclusion. It is very important.

In all, the current study focuses on the diachronic changes of MDD and NDD based on news and magazine texts from the COCA database. It shows that, rather than demonstrating a general trend of dependency distance minimization, these two text types witness great fluctuations over time. Besides that, the authors furthered their discussion in terms of the synergetic relations between syntactic complexity and communication efficiency. The authors not only show the quantitative results of the analyses, but also verifies the findings with statistical tests. However, there are still some points that need to be modified as I mentioned above. I sincerely hope these comments will help during the revision process.
