# Supplementary material for: An investigation of the diachronic trend of dependency distance minimization in magazines and news
Source: PLoS One. 2023 Jan 5;18(1):e0279836. doi: 10.1371/journal.pone.0279836 (PMC9815589; doi:10.1371/journal.pone.0279836)
Supplement: Attachment — Submitted filename: renamed_8cbfd.docx [file pone.0279836.s003.docx]

Dear editor Claudia Felser,

On behalf of my co-authors, we thank you very much for giving us an opportunity to revise our manuscript. We appreciate editor and reviewers very much for their positive and constructive comments and suggestions on our manuscript entitled “An investigation of the diachronic trend of dependency distance in news and magazines”（PONE-D-22-24114）.

We are very sorry to update the revised manuscript so late because the adding of some necessary supporting data. Especially we have to research related concept of information entropy which is very new to us.

In this revised version, we have addressed the concerns of the reviewers. An item-by-item response to the reviewers' comments is enclosed, and the revision was marked in red fronts in the manuscript. We hope that these revisions successfully address their concerns and requirements and that this manuscript will be accepted.

Looking forward to hearing from you soon.

Warm Regards

Guijun ZHOU

PhD, Professor of Linguistics, Northeast Normal University, PRC

Rebuttal

Reviewer #1:

The study closely followed Lei and Wen (2020), and examined the dependency distance minimization in a diachronic corpus of news and magazine texts. It is well designed and clearly written. I would suggest publication after the following issues are addressed.

1. While the study is a close replication of Lei and Wen (2020) and much similar results were obtained, significance of the study should be offered.

■ Lei and Wen (2020) used a much smaller dataset with no comparison between genres. The present study used much larger datasets and compared different text types of magazines and news, expanded the inspection scope without limiting sentence length. This is stated in:

*Scholars used mean dependency distance (MDD) and Normalized Dependency Distance (NDD) as indicators and based on the study of the diachronic dependence distance change of the State of the Union address in the United States, it was concluded that the dependence distance experience decreased over a long period [9]. This study, as the initial study on the diachronic change of the dependency distance, only examined the text type of the State of the Union addresses and the amount of data was relatively small. A subsequent study on other text types revealed that the dependency distance of different text types increased over time [10]. However, only MDD was used as an indicator, and the scope of the investigation was limited to sentences within a length of 10-30 words, which is not comprehensive enough to form a general idea of a certain tendency.*

*The present study explored the diachronic changes of dependency distance in different text types of magazines and news, as well as with larger datasets and expanded the inspection scope without limiting sentence length, and accordingly added the NDD inspection indicator, which is different from the MDD indicator because NDD takes into consideration the sentence length factor and the root word location factor in addition to the MDD [22].*

2. The authors stated that "This research also indicated that news text could be a text type that best balances language complexity and communication efficiency." in the abstract. However, I did not see anything related to this important argument, particularly those pertinent to "communication efficiency" in the body of the article.

■ I have revised the wording as “news text balances language complexity and communication efficiency better than magazine text”. A further explanation based on information entropy is newly provided in:

*With the assistance of the robust notion of* *information entropy [26], which can evaluate the information content as bits per character in written text [27], we adopted this algorism and carried it out on the sampled text of magazine and news text aforementioned. For two sentences with the same length of characters, the one with higher information entropy carries more information content within, which indicates a higher efficiency of information transmitting.*

| ***Information*** ***Entropy*** | ***Value*** |
| --- | --- |
| *Bits per Character for Magazines* | *15.30* |
| *Bits per Character for News* | *14.69* |

*Table 5 Information Entropy Comparison between Magazines and News Text*

*Thus it becomes clear that the magazines text while maintaining higher language complexity with* *a mean value of MDD of 2.86 ( NDD of 1.27 ), which is 1.09 times of* *the mean value of MDD of 2.61 ( 1.06 times of the mean value of NDD of 1.20 ) for news text, arrives at 15.30 bits per character of* *information entropy, only 1.04 times of the information entropy of 14.69 for news text. The benefit of complexity increase does not bring as much benefit of transmitting more information, which concludes that the news text better balances language complexity and communication efficiency than magazine texts.*

Reviewer #2:

**An investigation of the diachronic trend of dependency distance minimization in news and magazines**

This study quantitatively investigates the principle of dependency distance minimization (DDM) based on COCA. With special attention paid to the genres of news and magazines, it was found that both of these two text types witnessed some fluctuations diachronically in terms of the index of MDD and NDD, especially the text type of news. Moreover, the present study suggests that news texts achieve a balance between language complexity and communication efficiency.

Generally, the current work makes some exploration into the diachronic changes of the memory burden of these two text types. I appreciate that the authors make a good explanation of the theoretical background of the research and demonstrate their results clearly and concisely. However, one weak point of the work is the insufficient explanations of the reasons for the fluctuation of MDD and NDD with more references and citations to reach the conclusion that the text type of news cherishes a balance between complexity and efficiency.

Anyway, the article is a typical quantitative linguistic study that brings new findings to the diachronic changes of dependency distance and new insights into how MDD/NDD comes into force on different text types.

**I recommend accepting this study but the comments, which I itemize below, might be taken into consideration.**

**Comments on some specific sections:**

**Title**

- It might be better to change the title from “An investigation of the diachronic trend of dependency distance in **news and magazines**” to “An investigation of the diachronic trend of dependency distance in **magazines and news**”, since in the **Results and Discussion** part, the analysis of magazines are presented first, and then magazines. Also, please check through the whole text the order of “magazines” and “news”, including Tables.

■ Revised accordingly.

**Abstract**

- Line 10: “inspection indicators of MDD and NDD” > please give the full name of MDD and NDD in the abstract since they are not so familiar to the general readers of PLOSone.

■ Revised accordingly.

**Section 1 Introduction**

- Line 102: “language, compared with the dependency distance of languages randomly generated” > two spaces between “languages” and “randomly” should be deleted.

■ Revised accordingly.

- Line 101-159: The author grouped quantitative dependency studies into 7 different types. However, it is not so clear in terms of the differences among so many groups. I recommended dividing this part into different themes. For example, it can be divided into the factors that may affect dependency distance (e.g., Jiang and Liu 2015; Wang and Liu 2017; Yan and Liu 2021), the mechanism of dependency distance minimization and its impact (e.g., Liu 2007; Lu, Xu and Liu 2016), and the application of dependency measures and quantitative features of dependency structures (e.g., Liu, Zhao & Li 2009; Liu and Xu 2012; Jing and Liu 2015). Then, it comes to the lead-in part of the topic of the current research.

■ Revised accordingly. Though the “application of dependency measures and quantitative features of dependency structures” was divided into the other two. And another theme of “relationship between the principle of minimization of dependency distance, working memory, and cognition” is added.

**Section 2 Research Methods**

- Line 182-183: change the font of Table 1 to be consistent with the whole text or the format requirement of PLOSone.

■ Revised accordingly.

- Line 213: add a citation to “Standard CoreNLP 4.4.0”, e.g., Manning et al. 2014 (maybe).

■ Revised accordingly.

- Line 221-223: “Furthermore, considering the huge volume of the COHA corpus, the author randomly selected 30% of the original corpus for analysis and generated an annotated corpus for data analysis based on the randomly sampled text.” > Add information on the final raw data adopted after the data pre-processing and random selection. A table similar to Table 1 might be favorable.

■ Added accordingly.

- Line 224-225: “the average dependency distance MDD and the normalized average dependency distance NDD.” > (1) The expression should be reformulated. e.g., “the average dependency distance (MDD) and the normalized average dependency distance (NDD)”; (2) the citation should be added after MDD (Liu 2008) and NDD (Lei and Jockers 2020).

■ Revised accordingly.

- Line 228: punctuation mistake (please check through the whole text): *the lazy* *dog,” > dog.”,*

■ Revised accordingly.

- Line 232: In Table 2, row 1, column 2, “Parent node word(governor)” > “Parent node word (governor)”

■ Revised accordingly.

- Line 242: “normalized NDD” > the “N” means normalized.

■ Revised accordingly.

- Line 247: “MDD values can still be relied upon with large data sets”> two spaces between “values” and “can” need to be deleted.

■ Revised accordingly.

- Line 251: “The corresponding numerical values calculated are significantly” > two spaces between “values” and “calculated” need to be deleted.

■ Revised accordingly.

- Line 252: “Pearson correlation coefficient” > please check the assumptions of Pearson correlation. If the assumptions are not met, please use Spearman correlation.

■ I have researched into the difference between Pearson and Spearman, considering that the sampled years for whole text analysis are not sequenced, the Pearson correlation is favored.

- Line 252: 0.922 > please explicit the range of high, medium and low coefficients, respectively.

■ Revised accordingly.

**Section 3 Results and Discussion**

- Line 267: the present author > the author or the present study

■ Revised accordingly and also through the whole text.

- Line 274: R² > italicize statistics (*R²*)

■ Revised accordingly and also through the whole text..

- Line 280-281: “The goodness of fit of MDD and NDD for magazine texts is high” > what is “high”? Give the value ranges for good, medium and low coefficients of R square.

■ Revised accordingly.

- Line 320-335: The discussion is interesting and provoke-thinking. However, one very important shortcoming of this part is that more information on the features of news and magazines, especially more explanations of the characteristics of the news text type to reach a balance between syntactic complexity and communication efficiency, is highly needed.

■ I have revised the wording as “news text balances language complexity and communication efficiency better than magazine text”. A further explanation based on information entropy is newly provided.

**Section 4 Conclusion**

- Line 341-342: “Not all text types can reflect the diachronic dependency distance minimization trend” > this sentence is a little bit strange. You may delete it or add more explanations to this sentence.

■ Deleted

- Line 347: “ the upper limit of the dependence distance as 3” > should it be 4, rather than 3? Please check it and add a citation.

■ Citation added.

- Line 359: “could be regarded as mass media. [27]” > wrong placement of the punctuation mark, it should be “could be regarded as mass media [27] .”

■ Revised accordingly.

- Line 359-361: “The research results have revealed that the language complexity of magazines, elite media, has now approached that of mass media.” > add more explanation of this sentence in **Results and Discussion** so as to reach this conclusion. It is very important.

■ Revised accordingly. For details please see:

*When comparing the MDD and NDD of magazines texts and news text together, it can be noticed that the lowest point of the general downward trend of magazine texts coincides with the news texts , implying that the easy-to-understand news texts marks a certain boundary of the decreasing trend of dependence distance, or rather, the dependency distribution for magazines texts had been dropping closer to that of the news texts continuously in the past years.*

*This can also help to explain the observed trend in previous discussion that the trend of MDD and NDD of magazines texts are coming closer to that of news text in later years among the collected data. After all, the principle of labor-saving is to achieve the purpose of communication. The language of news is to a large extent the language that readers are most familiar with, and using familiar language in news helps to determine the credibility of the people and things on the newspaper page [28].*

In all, the current study focuses on the diachronic changes of MDD and NDD based on news and magazine texts from the COCA database. It shows that, rather than demonstrating a general trend of dependency distance minimization, these two text types witness great fluctuations over time. Besides that, the authors furthered their discussion in terms of the synergetic relations between syntactic complexity and communication efficiency. The authors not only show the quantitative results of the analyses, but also verifies the findings with statistical tests. However, there are still some points that need to be modified as I mentioned above. I sincerely hope these comments will help during the revision process.

\# Claudia Felser, Ph.D Academic Editor

Although the reviewers were both essentially positive about your manuscript, they have made several useful suggestions for improvement. Among other things they ask you to make the significance of your findings clearer, in particular in relation to the findings reported in Lei and Wen's (2020) study. They also ask for an improved discussion of the difference between the text types you examined, and of how news texts manage to balance complexity and efficiency. Reviewer 2 also makes a number of other suggestions that you should consider in your revisions.
I also have some additional comments of my own, which I am asking you to address in your revisions.
1. The abstract should be revised and re-focused. At present it focuses on Lei's work but says very little about the current study. The abstract also contains some unexplained abbreviations (MDD and NDD).

■ Abbreviations explained and abstract revised accordingly.
2. The introductory part contains several unclear wordings and attributions. E.g.

■Introductory part redesigned as proposed by reviewer 2.
l.102 "One direction..." What do you mean here? Direction of current research?

■Direction of previous research. *Previous* added.
l.110 "The study..." - What study are you referring to here?

■ Citation comes at the end of the sentence.
l.117 "in the monolingual corpus and the hybrid corpus..." - What corpora are these? They have not been mentioned before. And what is meant by "hybrid"?

■Citation comes at the end of the sentence. "hybrid" refers to a corpus in which, for example, a line of sentence contains both English and Chinese, which brought influence upon the dependency structure.
l.132 "young people's" - Is this finding specific to young people, and why do young people specifically matter for your study?

■ For the referenced article, they take young perple as data collecting target as they withhold normally the best memory capability, which helps when examine the upper limit of working memory. For the current reaearch , it is not limited on young people.
l.148 "The last aspect of the relevant study..." - What study are you referring to here?

■ Wording revised accordingly.
3. Please make sure that all figures and tables are explicitly referred to in your main text.

■ Revised accordingly.
4. Your title should be changed so as to include the term "dependency distance minimization" or "minimization of dependency distance". The current wording "diachronic trend of dependency distance" is uninformative.

■ Revised accordingly.
